# Long noncoding RNA expression profile in HLE B-3 cells during TGF-β_2_-induced epithelial-mesenchymal transition

**DOI:** 10.1186/s12886-017-0461-z

**Published:** 2017-05-16

**Authors:** Bingyu Zhang, Yang Chen, Meiyuan Qiu, Zhixiang Ding

**Affiliations:** 1 Guilin Medical University, Guangxi Zhuang Autonomous Region, Guilin, 541000 China; 2 Department of Ophthalmology, Guilin Medical University Affiliated Hospital, Guangxi Zhuang Autonomous Region, Guilin, 541001 China

**Keywords:** LncRNA, Microarray, HLE B-3 cells, Epithelial-mesenchymal transition, Posterior capsule opacification

## Abstract

**Background:**

Recent evidence has shown that long noncoding RNAs (lncRNAs) are involved in the process of epithelial-mesenchymal transition (EMT). However, little research has focused on the expression profile of lncRNAs during EMT in human lens epithelial cells (LECs) and their functions have not yet been described.

**Methods:**

Dysregulated lncRNAs and mRNAs in normal human lens epithelial B-3(HLE B-3) cells and during transforming growth factor β_2_(TGF-β_2_)-induced EMT were analyzed via lncRNA microarray. Gene Ontology (GO) and Kyoto Encyclopedia of Genes and Genomes (KEGG) Pathway analyses of differentially expressed mRNAs were performed to identify their functions and pathologic pathways. Six candidate lncRNAs were validated via quantitative real-time reverse transcription polymerase chain reaction(qRT-PCR) to confirm the microarray data.

**Results:**

A total of 775 lncRNAs (325 up-regulated and 450 down-regulated) and 935 mRNAs (329 up-regulated and 606 down-regulated) were differentially expressed in HLE B-3 cells during TGF-β2-induced EMT compared to normal HLE B-3 cells. GO and KEGG Pathway analyses indicated the functions of differentially expressed mRNAs in the TGF-β_2_-induced EMT in HLE B-3 cells. qRT-PCR confirmed the trends indicated in microarray analysis for all 6 candidate lncRNAs.

**Conclusion:**

Our study lays the foundation for future research in lncRNAs related to EMT in HLE B-3 cells and could provide new avenues for the prevention and treatment of posterior capsule opacification (PCO).

## Background

Cataracts are the leading cause of blindness, and account for 51% of blindness, about 20 million people, and 33% of visual impairment worldwide, according to the latest assessment (2010) [1]. Surgery is an effective treatment for cataracts, however, complications can arise [2]. Posterior capsule opacification (PCO) is the most common complication after cataract surgery [3]. The residual lens epithelial cells (LECs) in the anterior capsule after cataract surgery, over proliferate, and migrate to the posterior capsule, and then epithelial-mesenchymal transition (EMT) takes place. This dynamic process is the main mechanism of PCO [4–6]. EMT is an abnormal differentiation of epithelioid cells to myofibroblast cells and is central to the development of PCO [7]. Inhibition of EMT has emerged as an option for treating PCO.

Transforming growth factor β_2_ (TGF-β_2_) is a signaling molecule, that plays a crucial role in activating and promoting EMT in LECs. Recent studies have shown that TGF-β_2_ can induce EMT in LECs through the Smads pathway, PI3K/Akt pathway, and other pathways [8, 9]. TGF-β_2_-induced EMT is a proper model for studying PCO [10].

Long noncoding RNAs (lncRNAs) are transcripts of more than 200 nucleotides, that do not have the ability to encode proteins, but participate in transcriptional, post-transcriptional and epigenetic regulation of gene expression [11, 12]. The expression, mechanism, and function of lncRNAs have attracted attention in recent years [13]. However, it’s not clear how lncRNAs are related to the TGF-β2-induced EMT in LECs.

To understand the functions of lncRNAs in TGF-β_2_-induced EMT in LECs, we performed lncRNA and mRNA microarray analysis in normal LECs and those in TGF-β_2_-induced EMT. Gene Ontology (GO) analysis [14, 15] and Kyoto Encyclopedia of Genes and Genomes (KEGG) pathway analysis [16, 17] were performed to identify the functions of differentially expressed genes. The results of our study indicate that dysregulated expression of lncRNAs may influence the development and progression of EMT in LECs and that studying these lncRNAs may introduce new avenues for the prevention and treatment of PCO.

## Methods

### Cell culture and treatment

The human lens epithelial B-3 (HLE B-3) cell line, a type of LEC, was purchased from the Institute of Biochemistry and Cell Biology, SIBS, CAS (Shanghai, China). It was cultured in Dulbecco’s modified Eagle’s medium (Gibco, GranIsland, NY, USA) supplemented with 15% fetal bovine serum (Gemini, West Sacramento, CA, USA). Cells were maintained in humidified air with 5% CO_2_ at 37 °C before use. For further experiments, cells were trypsinized and seeded in 6 wells plates. When cell cultures reached 70% confluence, 3 wells of cells were stimulated with 10 ng/ml recombinant human TGF-β_2_ (Peprotech, Inc., Rocky Hill, NJ, USA) for 24 h, while the other 3 wells of cells were incubated in DMEM for 24 h. Following treatment, phase-contrast micrographs were used to observe morphology change of cells. Then the cells were collected for quantitative real-time reverse transcription polymerase chain reaction (qRT-PCR), western blot analysis, and immunofluorescence assay.

### qRT-PCR of cells

Total RNA was extracted using TRIZOL Reagent (Cat#15596-018,Life Technologies, Carlsbad, CA, US), and then reverse transcribed using Prime Script RT Master Mix (TaKaRa, Dalian, China) according to the manufacturer’s instructions. qRT-PCR was performed using SYBR Green PCR Master Mix (ABI Applied Biosystems, Foster City, CA, USA) in the ABI 7900HT sequence detection system (ABI Applied Biosystems, Foster City, CA, USA) following the manufacturer’s instructions, and beta-actin was used as an internal control. All the genes were amplified in separate wells in triplicate. The following primer pairs were used: E-cadherin (F 5′- AGCAGAACTAAACACACGGGG -3′, R 5′- ACCCA CCTCTAAGGCCATCT -3′), vimentin (F 5′- GACGCCATCAACACCG AGTT-3′, R 5′- GTTTGTCGTTGGTTAGCTGGT -3′), α-SMA (F 5′- GTGTTGCCCCTGAAGAGCAT -3′, R 5′- GCTGGGACATTGAAAGT CT CA -3′), and beta-actin (F 5′- CTGGAACGGTGAAGGTGA CA -3′, R 5′- CGGCCACATTGTGAACTTTG -3′). Gene expression was calculated using the 2^-ΔΔ^CT method [18].

### Western blot

The cells were collected and lysed in lysis buffer on ice, and the proteins were quantified using a Pierce BCA Protein Assay kit (Thermos Fisher Scientific, Inc., Waltham, MA, USA). Cell lysates were separated by 8-12% sodium dodecyl sulfate polyacrylamide gel electrophoresis, and proteins were transferred to polyvinylidene difluoride membranes. The membranes were subsequently incubated with the following primary antibodies overnight at 4 °C: E-cadherin (1:1000; cat. no. 3195; Cell Signaling Technology, Inc.), α-SMA (1:1000; cat. no. 4691; Cell Signaling Technology, Inc.), and vimentin (1:1000; cat. no. 5741; Cell Signaling Technology, Inc.). The membranes were washed three times with TBS/T and then incubated for 1 h in IRDye®680RD goat anti-rabbit immunoglobulin G (H + L) diluted at 1:5000 in TBST. Protein levels were visualized and quantified using the LI-COR Odyssey scanner and software (LI-COR Biosciences).

### Immunofluorescence assay

After specific treatment, HLE B-3 cells were fixed with ice-cold methanol for 10 min. After washed with PBS for three times, non-specific binding sites were blocked with 4% bovine serum albumin for 30 min at room temperature. Cells were incubated with the primary antibodies (E-cadherin, 1:100; cat. no. 3195; Cell Signaling Technology, Inc. α-SMA, 1:100; cat. no. 4691; Cell Signaling Technology, Inc. vimentin, 1:100; cat. no. 5741; Cell Signaling Technology, Inc.) overnight at 4 °C, and then incubated with the FITC-conjugated secondary antibody for 1 h at room temperature. After counterstained with 4,6-diamidino-2-phenylin-dole (DAPI) for 3 min, these cells were subsequently observed under a confocal microscope(Carl Zeiss, LSM710, Jena, Germany).

### Microarray

The microarray (SBC Human lncRNA microarray v6.0, Shanghai Biotechnology Corporation, Shanghai, China) used in this study detects approximately 77,103 lncRNAs and 18,853 coding transcripts. The lncRNAs were carefully constructed using the most highly respected public transcriptome databases including Ensembl (http://www.ensembl.org/index.html), LNCipedia (http://www.lncipedia.org/), Lncrnadb (http://lncrnadb.org/), Noncoder (http://www.noncode.org/), and UCSC (http://genome.ucsc.edu/index.html). 6 sets of microarray assay were done, including 3 for the control group, and 3 for the TGF-β2 group.

### RNA extraction

Total RNAs were extracted using TRIZOL Reagent (cat. No. 15596-018, Life Technologies, Carlsbad, CA, US) following the manufacturer’s instructions, and the RNA integrity number was calculated using an Agilent Bioanalyzer 2100 (Agilent Technologies, Santa Clara, CA, US). Qualified total RNA was further purified via an RNeasy micro kit (cat. No. 74004, QIAGEN, GmBH, Germany) and RNase-Free DNase Set (cat. No. 79254, QIAGEN, GmBH, Germany).

### RNA labeling and array hybridization

Total RNA was amplified and labeled using a Low Input Quick Amp WT Labeling Kit (cat. no. 5190-2943, Agilent Technologies, Santa Clara, CA, US), according to the manufacturer’s instructions. Labeled cRNAs were purified using an RNeasy mini kit (cat. no. 74106, QIAGEN, GmBH, Germany).

Each slide was hybridized with 1.65 μg Cy3-labeled cRNA using a Gene Expression Hybridization Kit (cat. no. 5188-5242, Agilent Technologies, Santa Clara, CA, US) in a Hybridization Oven (cat. no. G2545A, Agilent Technologies, Santa Clara, CA, US). After 17 h of hybridization, slides were washed in staining dishes (cat. no. 121, Thermos Shandon, Waltham, MA, US) with a Gene Expression Wash Buffer Kit (cat. no. 5188-5327, Agilent Technologies, Santa Clara, CA, US).

### Data analysis

Slides were scanned with an Agilent Microarray Scanner (cat. no. G2565CA, Agilent Technologies, Santa Clara, CA, US). Data were extracted with Feature Extraction software v10.7 (Agilent Technologies, Santa Clara, CA, US). Raw data were normalized using the Quantile algorithm, GeneSpring software v12.6.1 (Agilent Technologies, Santa Clara, CA, US). Fold Change filtering and Student’s t test were used to identify differentially expressed lncRNAs and mRNAs (fold change ≥2.0, *P* < 0.05).

### Gene function analysis

To identify the functions of lncRNAs, target genes of differentially expressed lncRNAs were predicted via cis- or trans-regulatory effects. GO analysis was performed to analyze the functions of differentially expressed mRNAs by using the Database for Annotation, Visualization, and Integrated Discovery (http://david.abcc.ncifcrf.gov/) [14, 15]. Pathway analysis was used to determine the significant biological pathways of these differentially expressed mRNAs according to KEGG, (http://www.genome.jp/eg/) [16, 17]. The threshold of significance was defined by the *P*-value (recommended *P* < 0.05).

### qRT-PCR validation

qRT-PCR was used to validate the microarray data. The method was the same as that described in section "qRT-PCR of cells". The following primer pairs were used: NR-015410 (F 5′- CTGTCTAATTTTCCAGAGCCCCT -3′, R 5′- GTCATCTCTCCCCCA CATACC -3′), ENST00000618591 (F 5′- GGGAGAGCATTCTTCCAG GT -3′, R 5′- GGACACTGTGAACGGAGACA -3′), ENST00000512323 (F 5′- ATCTGCACTGGTGTGAGGTTT -3′, R 5′- GTACTGCTCTTCCTGGTGCTG -3′), ENST00000528717 (F 5′- GTTT CTTGGAATGTGAAAGTCG -3′, R 5′- CCATAGGCAGTAGTAGCCC AAC -3′), lnc-PF4-1:1 (F 5′- AACTGCCTTGCCAGTGCTT -3′, R 5′- A GGGGACTTCACGTTCACAC -3′), NR-034138 (F 5′- TTGGAAGAAT CCTGGAAGCA -3′, R 5′- CAGAAGAAAGAGACCCTCATGG -3′), and beta-actin (F 5′- CTGG AACGGTGAAGGTGACA -3′, R 5′- CGGCCA CATTGTGAACTTTG -3′). Gene expression was calculated using the 2^-ΔΔCT^ method.

### Statistical methods

All statistical data were analyzed using SPSS software v18.0 s (SPSS Inc., Chicago, IL, USA). The threshold value we used to screen differentially expressed lncRNAs and mRNAs was a fold change ≥2.0 (*P* < 0.05). LncRNAs and mRNAs expressed differentially in the TGF-β_2_ group compared to the control group were analyzed using Student’s t tests. *P* < 0.05 was considered statistically significant. The false discovery rate was calculated to correct the *P*-value.

## Results

### QRT-PCR/Western blot/Immunofluorescence of cells

**Fig. 1:**
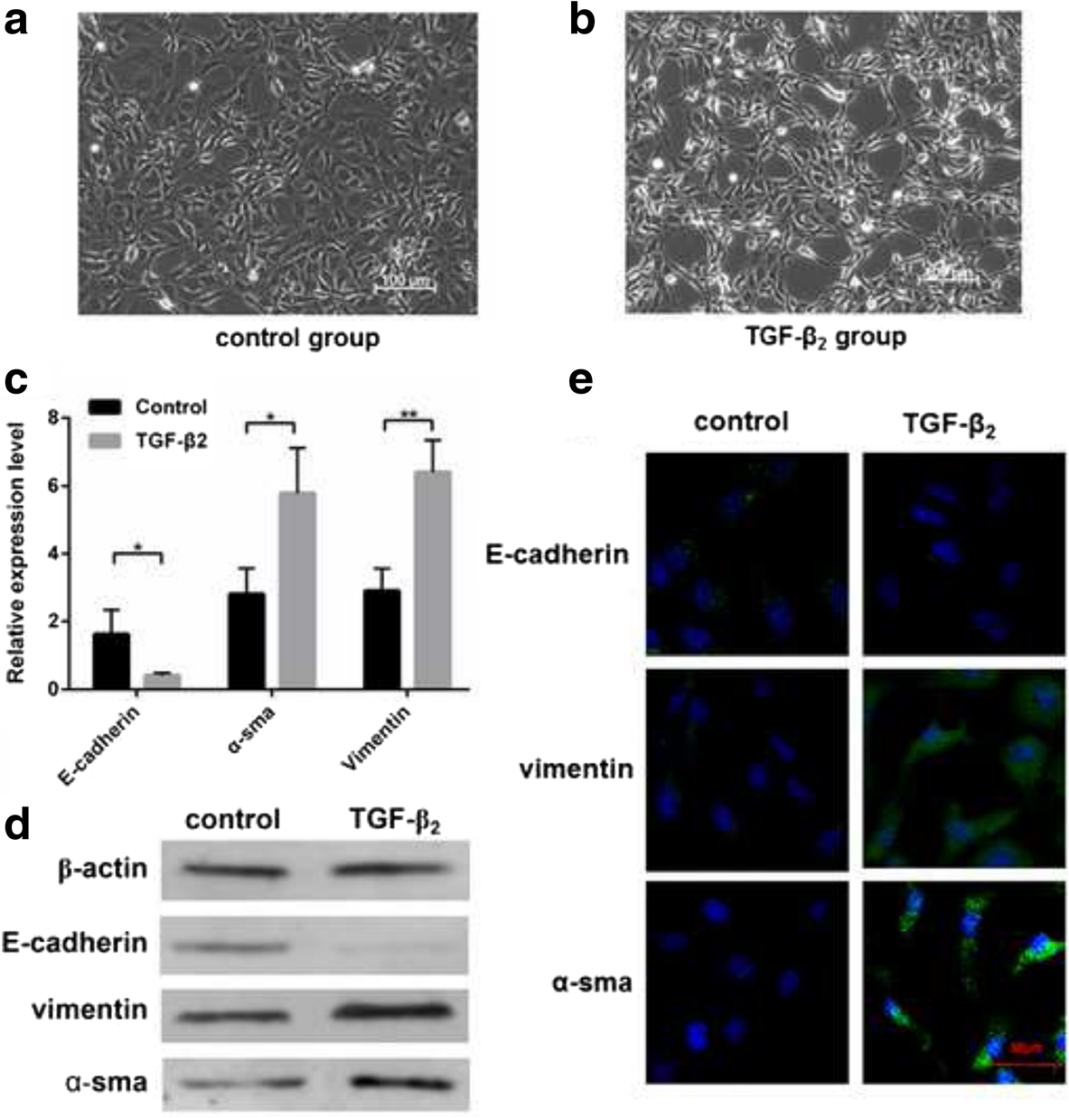
TGF-β_2_ group cells compared to control group cells. Phase-contrast micrographs were used to observe cells in the control group (**a**) and TGF-β_2_ group (**b**). The single polygonal cells changed into long, spindle-shaped cells. **c** Total RNAs were extracted from the control group(*n* = 3) and TGF-β_2_ group(*n* = 3), qRT-PCRs were conducted to detect the expression of E-cadherin, α-SMA, and vimentin(*P < 0.05, **P < 0.01). **d** The proteins were collected from the control group(*n* = 3) and TGF-β_2_ group(*n* = 3), western blot was conducted to detect the expression of E-cadherin, α-SMA, and vimentin. **e** The expression of E-cadherin, α-SMA, and vimentin were detected by immunofluorescence

Phase-contrast micrographs showed, after stimulated with 10 ng/ml TGF-β_2_, cells were transformed from single polygonal to long and spindle-shaped (Fig. 1). To confirm EMT in TGF-β_2_ group, we detected 3 typical EMT-related biomarkers including E-cadherin, α-SMA, and vimentin [19] by qRT-PCR, Western blot, and immunofluorescence. All experiments showed that E-cadherin was reduced, while α-SMA and vimentin expression was increased (Fig. 1c-e). In conclusion, HLE B-3 cells in TGF-β_2_ group were undergoing EMT.

### Differentially expressed lncRNAs and mRNAs

**Fig. 2:**
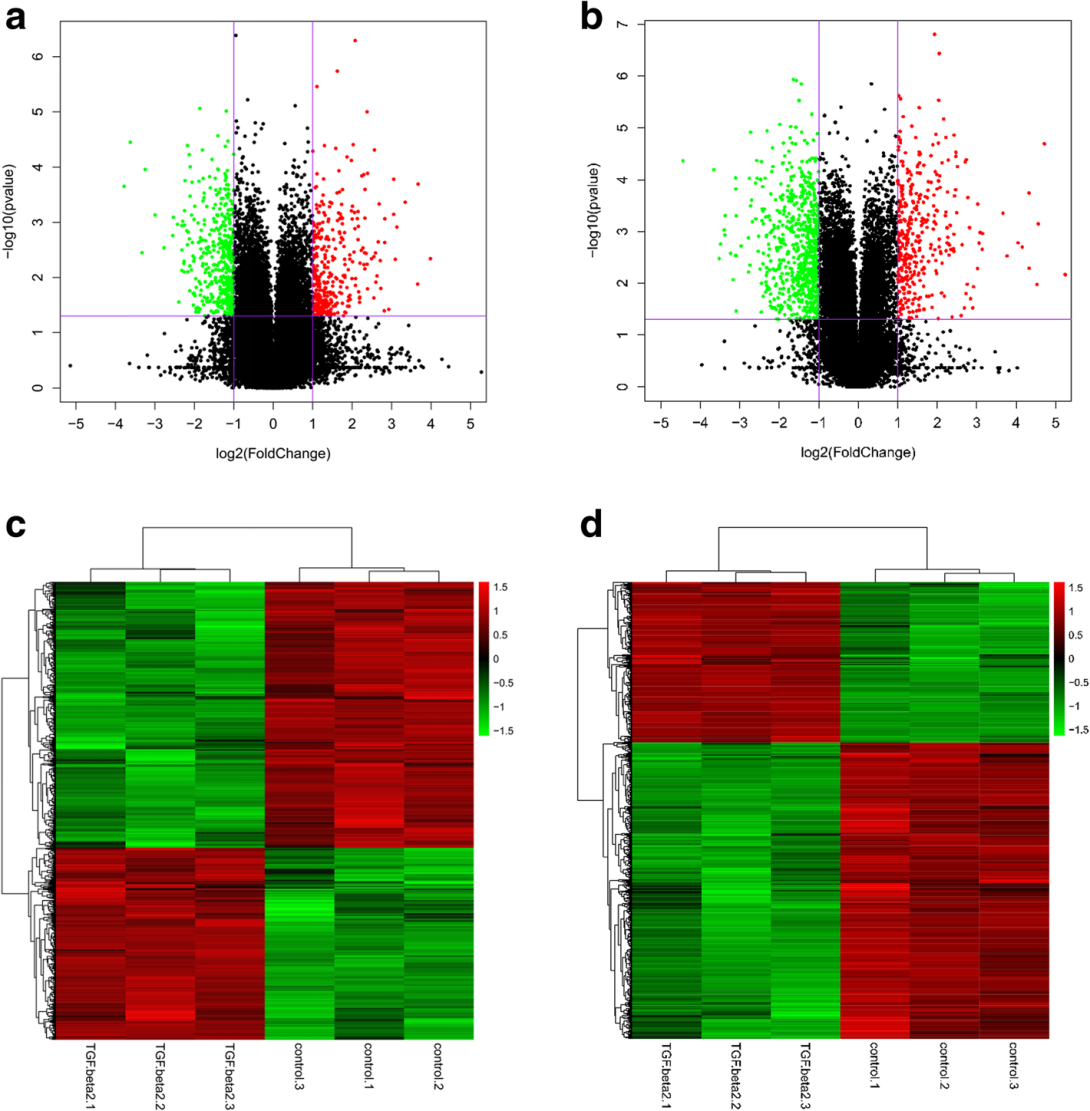
Gene expression profile differences between the TGF-β_2_ group compared to the control group. Volcano plots were used to distinguish the differentially expressed lncRNAs (**a**) and mRNAs (**b**). The vertical lines correspond to 2.0-fold up and down and the horizontal line represents a *P* value of 0.05. Hierarchical clustering indicates lncRNAs (**c**) and mRNA (**d**) profiles. Red and green indicate high and low expression, respectively. In the heat map, the columns represent samples, and the rows represent RNAs

To screen the differentially expressed lncRNAs and mRNAs with statistical significance (fold change ≥2.0, *P* < 0.05) between TGF-β_2_ group and control group, volcano plots were constructed (Fig. 2a, b). Furthermore, the lncRNA and mRNA expression patterns across the samples were distinguishable in the heat map generated by hierarchical clustering (Fig. 2c, d).

Based on the microarray data, 775 lncRNAs were differentially expressed (fold change ≥2.0, *P* < 0.05), including 325 upregulated lncRNAs and 450 downregulated lncRNAs. The top 20 upregulated lncRNAs and top 20 downregulated lncRNAs between the two groups are listed in Table 1. Lnc-PMEPA1-2:1 (fold change: 15.830) was the most significantly upregulated lncRNA, and NR-033931 (fold change: 13.756) was the most significantly downregulated lncRNA. Downregulated lncRNAs were found to be more common than upregulated lncRNAs in these microarray data.

**Table 1:** Top 20 differentially expressed lncRNAs in TGF-β_2_ group compared to control group

Up-regulated lncRNAs				Down-regulated lncRNAs			
LncRNA	Source	*p*-values	Fold change	LncRNA	Source	*p*-values	Fold change
lnc-PMEPA1-2:1	Lncipedia	0.00455517	15.830	NR_033931	RefSeq	0.00022164	13.756
ENST00000597865	GENCODE	0.00020187	12.737	ENST00000582120	GENCODE	0.00308787	12.854
lnc-ZNF737-1:1	Lncipedia	0.01317849	12.647	lnc-FAIM3-2:1	Lncipedia	3.51412E-05	12.313
lnc-PCK1-2:1	Lncipedia	0.00250698	12.061	ENST00000580242	GENCODE	0.00355059	10.021
lnc-RIOK2-5:1	Lncipedia	4.6625E-06	10.882	NR_110260	RefSeq	0.00718676	9.901
lnc-ABCA12-5:2	Lncipedia	0.00043109	10.147	ENST00000508352	GENCODE	0.00010915	9.486
lnc-SMAD5-7:1	Lncipedia	1.89988E-05	9.649	lnc-SIX3-3:1	Lncipedia	0.00093703	8.399
ENST00000558888	GENCODE	0.00121548	8.765	NR_033888	RefSeq	0.00173551	5.851
ENST00000606197	GENCODE	0.00469381	8.533	NR_033931	RefSeq	0.00080852	5.791
ENST00000591217	GENCODE	0.00195645	8.503	lnc-PSG6-3:1	Lncipedia	0.00369578	5.391
NR_125383	RefSeq	0.00016452	8.302	ENST00000562459	GENCODE	0.00108506	5.383
NR_033957	RefSeq	0.00071386	8.289	ENST00000513853	GENCODE	0.02789757	5.259
ENST00000540392	GENCODE	0.00051516	7.495	NR_110294	RefSeq	0.02014080	5.164
ENST00000603720	GENCODE	0.00085945	7.098	NR_038929	RefSeq	0.00114790	4.993
lnc-ABCA12-6:1	Lncipedia	0.00232021	7.083	NR_049793	RefSeq	0.00317515	4.978
ENST00000508884	GENCODE	0.00243957	6.931	NR_027995	RefSeq	0.00158735	4.929
ENST00000557900	GENCODE	0.00351377	6.719	lnc-PSG4-1:1	Lncipedia	0.00025116	4.855
ENST00000606197	GENCODE	0.01581059	6.615	ENST00000508352	GENCODE	0.00557057	4.814
NR_015410	RefSeq	0.00494265	6.552	ENST00000605586	GENCODE	0.00670910	4.800
NR_109998	RefSeq	0.00169954	6.531	lnc-ABCD3-1:2	Lncipedia	0.00791576	4.786

Using the same criteria for lncRNAs, we identified 935 mRNAs that were differentially expressed (fold change ≥2.0, *P* < 0.05), including 329 upregulated mRNAs and 606 downregulated mRNAs. The top 20 upregulated and top 20 downregulated mRNAs between the two groups are listed in Table 2. C4orf26 (fold change: 37.775) was the most significantly upregulated mRNA, and KRTAP1-5 (fold change: 21.691) was the most significantly downregulated mRNA.

**Table 2:** Top 20 differentially expressed mRNAs in TGF-β_2_ group compared to control group

Up-regulated mRNAs			Down-regulated mRNAs		
mRNA	*p*-values	Fold change	mRNA	*p*-values	Fold change
NM_001206981(C4orf26)	0.00682992	37.775	NM_031957(KRTAP1-5)	4.30506E-05	21.691
NM_004118(FOXS1)	2.01224E-05	26.221	NM_004657(SDPR)	6.36942E-05	12.608
NM_020182(PMEPA1)	0.00070997	23.537	NM_002974(SERPINB4)	0.00334950	11.493
NM_020182(PMEPA1)	0.01058581	23.038	NM_001870(CPA3)	0.00175241	11.208
NM_001001557(GDF6)	0.00513492	20.032	NM_000740(CHRM3)	0.00092973	10.537
NM_000399(EGR2)	0.00018028	20.013	NM_005130(FGFBP1)	0.00116285	10.382
NM_001855(COL15A1)	0.00199402	17.745	NM_020949(SLC7A14)	0.00049138	10.333
NM_212557(AMTN)	0.00165480	16.538	NM_006727(CDH10)	0.00264798	9.407
NM_182908(DHRS2)	0.00296753	13.614	NM_006495(EVI2B)	0.00850874	8.984
NM_001145320(ADAMTSL2)	0.00044198	12.692	NM_145260(OSR1)	9.19462E-05	8.614
NM_001142393(NEDD9)	0.00109819	8.843	NM_001261461(NFE2)	0.00014986	8.590
NM_020400(LPAR5)	0.00220825	8.816	NM_001165252(KRTAP2-3)	0.00291367	8.573
NM_001135057(LRRC15)	0.00104638	8.510	NM_000891(KCNJ2)	0.00049496	8.531
NM_014443(IL17B)	0.00166941	8.333	NM_001287746(HMGCLL1)	0.00191632	7.812
NM_001781(CD69)	0.00029504	8.139	NM_033317(DMKN)	0.00132214	6.892
NM_000888(ITGB6)	0.00518906	8.100	NM_006512(SAA4)	0.00609439	6.853
NM_004717(DGKI)	0.02999408	7.453	NM_130386(COLEC12)	9.35347E-05	6.760
NM_001955(EDN1)	0.01998286	7.174	NM_002164(IDO1)	0.00446529	6.698
NM_022166(XYLT1)	0.00084475	7.043	NM_014033(METTL7A)	0.00031852	6.623
NM_000023(SGCA)	0.00022045	6.844	NM_001160354(LY6K)	1.20466E-05	6.614

### LncRNA target prediction

To identify the potential function of differentially expressed lncRNAs, we predicted the target genes by target prediction programs. 565 lncRNAs had cis target genes, and 213 lncRNAs had trans genes.

### GO and KEGG pathway analysis of differentially expressed mRNAs

**Fig. 3:**
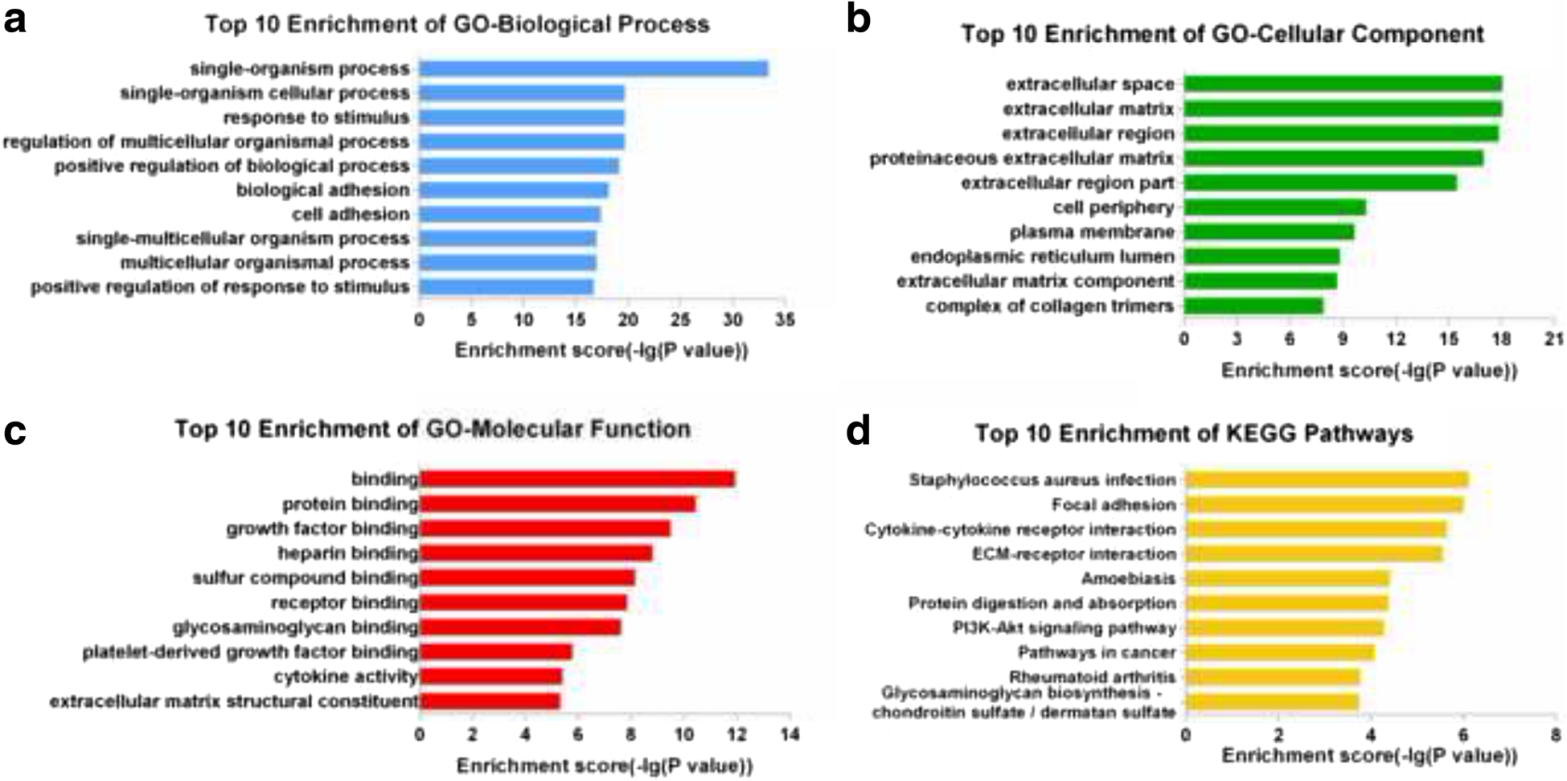
Enrichment analysis of GO terms and pathways for differentially expressed mRNAs. GO analysis according to 3 GO categories: **a** biological process, **b** cellular component, and **c** molecular function. **d** Pathway analysis based on the KEGG database

To determine the potential roles of differentially expressed lncRNAs, GO analysis and KEGG pathway analysis were applied to the differentially expressed mRNAs. The GO categories comprised 3 structured networks: biological processes, cellular components and molecular function. We found the most enriched GO terms associated with differentially expressed mRNAs were “single-organism process” (biological process) (Fig. 3a), “extracellular space” (cellular component) (Fig. 3b), and “binding” (molecular function) (Fig. 3c).

KEGG Pathway analysis indicated that 36 pathways were significantly enriched among the differentially expressed mRNAs (*P* < 0.05) (Fig. 3d). The most enriched pathway was “*Staphylococcus aureus* infection”, which was associated with 13 differentially expressed genes. Many of these pathways were linked to EMT, such as the PI3K-Akt signaling pathway (associated with 32 genes), TGF-β signaling pathway (associated with 11 genes), ECM-receptor interaction (associated with 15 genes), regulation of actin cytoskeleton (associated with 17 genes), and cell adhesion molecules (associated with 15 genes).

### qRT- PCR validation

**Fig. 4:**
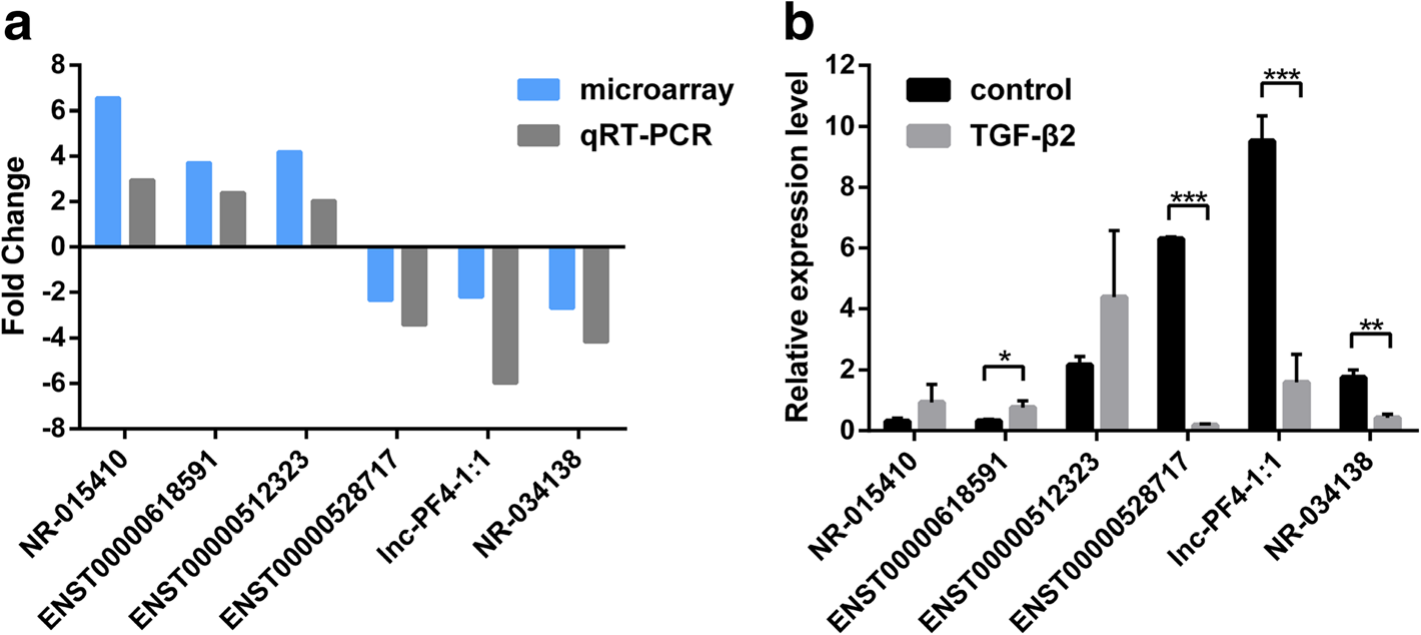
qRT-PCR validation of 6 differentially expressed lncRNAs. **a** Comparison of fold change of lncRNAs between microarray and qRT-PCR results. **b** The relative expression levels of lncRNAs in the control group and TGF-β_2_ group (*P < 0.05, **P < 0.01, ***P < 0.001)

To confirm the validity of the microarray data, we randomly selected 6 differentially expressed lncRNAs for qRT-PCR. These included 3 upregulated lncRNAs (NR-015410, ENST00000618591, and ENST00000512323) and 3 downregulated lncRNAs (ENST00000528717, lnc-PF4-1:1, NR-034138). qRT-PCR was carried out to confirm the expression of the selected lncRNAs in LECs during TGF-β_2_-induced EMT. qRT-PCR showed the same trend for the 6 lncRNAs that were shown in the microarray analysis (Fig. 4a). The changes were statistically different for only 4 of the 6 lncRNAs (Fig. 4b). ENST00000618591 was upregulated, while ENST00000528717, lnc-PF4-1:1, and NR-034138 were downregulated (*P* < 0.05).

## Discussion

LncRNAs are among the least well-understood of non-protein-coding RNAs. They were previously considered merely transcriptional “noise” [11] but have increasingly garnered attention in recent years. Newer studies have shown that lncRNAs are involved in EMT. For example, several lncRNAs can be involved in the regulation or activation of the WNT signaling pathway in the Twist-induced EMT process [20]. H19 can promote pancreatic cancer metastasis by derepressing let-7’s suppression on its target HMGA2-mediated EMT [21]. However, to our knowledge, no previous study has focused on the microarray expression profile of lncRNAs in LECs during EMT. Thus, we conducted the current study to assess the role of lncRNAs in the development and progression of EMT in LECs from the perspective of lncRNA.

In this study, we chose the HLE B-3 cell line. It is a primary cell line of LECs that is immortalized via infection with an adenovirus 12-SV40 virus, and can be used to investigate HLE physiology and cataracts [22]. We chose 3 typical biomarkers: E-cadherin, α-SMA, and vimentin to confirm EMT in LECs [19]. E-cadherin is the prototypical epithelial cell marker of EMT. It is expressed in epithelial cells, and its expression is decreased during EMT. Vimentin and α-SMA are mesenchymal markers, and their expression is increased during EMT [6, 7]. The results of qRT- PCR, western blot and immunofluorescence suggested that the cells in the TGF-β_2_ group were undergoing EMT.

The findings of our study indicate that lncRNAs play a potential role in the EMT pathogenesis of PCO. A total of 325 upregulated lncRNAs and 450 downregulated lncRNAs were differentially expressed in the TGF-β_2_ group compared to the control group. Furthermore, we used GO and KEGG pathway analyses to identify potential functions of the differentially expressed mRNAs. GO analysis revealed the dysregulation of 1165 mRNAs involved in biological processes, 72 mRNAs involved in cellular components, and 112 mRNAs involved in molecular functions. Many items were involved in critical processes in EMT, such as protein binding, growth factor binding, cell proliferation. KEGG Pathway analysis indicated that 36 pathways were significantly enriched, many of which are related to EMT, including the PI3K-Akt signaling pathway [8, 23], TGF-β signaling pathway, and ECM-receptor interaction [24, 25]. These results indicated the general functional roles of the differentially expressed lncRNAs and also confirmed the reliability of our microarray data.

Although huge numbers of lncRNAs have been found, the functions of most lncRNAs remain unknown. Previous reports have suggested that lncRNAs can guide gene expression either in cis (on neighboring genes) or in trans (on distantly located genes) manner [26, 27]. We can predict the functions of lncRNA via the cis/trans genes. We chose the top 4 up and down lncRNAs to analyze. The upregulated lnc-PMEPA1-2:1 is a 361 bp sense-overlapping lncRNA and was predicted to have a cis target gene, prostate transmembrane protein, androgen-induced 1(PMEPA1), which is important in cancer development. PMEPA1 encodes a transmembrane protein that contains a Smad-interacting motif [28]. PMEPA1 expression is induced by androgens and TGF-β, and can suppress the androgen receptor and TGF-β signaling pathways by interacting with Smad proteins. In breast cancer cells, PMEPA1 could be upregulated by classical TGF-β/Smad signaling pathway, and silencing of PMEPA1 significantly could inhibit the migration ability of MDA-MB-231 cells and promoted the process of EMT. Previous studies also showed that PMEPA1 can regulate EMT in lung cancer cells by modulating the ROS and IRS-1 signaling pathways [28]. Lnc-PMEPA1-2:1 may regulate EMT in PCO by PMEPA1 via the signaling pathways. NR-033931, also known as linc01085, is a 1984 bp intergenic lncRNA, of which little is known. ENST00000597865 is a 520 bp antisense lncRNA, and have a cis genes Neurotrophin 4(NTF4), which is a member of the nerve growth factor family of neurotrophins, that control survival and differentiation of mammalian neurons. Kim et al. reported that NTF4 receptor TrkB could induce EMT through activation of the JAK2/STAT3 pathway and PI3K/AKT pathway in breast cancer [29]. NTF4 may have the similar function in EMT of PCO. ENST00000582120 is a 673 bp sense lncRNA. It had a cis gene: collectin subfamily member 12 (COLEC12). COLEC12 is a scavenger receptor, a cell surface glycoprotein that displays several functions associated with host defense. According to the GO analysis, COLEC12 plays roles in biological process(single-organism process, response to stimulus, and positive regulation of biological process) and cellular component(extracellular region, cell periphery, and plasma membrane). ENST00000582120 may play roles in EMT via regulating COLEC12. Further researches are needed to confirm the functions of these differentially expressed lncRNAs and their potential target genes.

Despite these promising findings, the current study did have some limitations. First, the sample size in the microarray analysis was small, as was the number of candidate lncRNAs, and these might limit the validity of the array results. Second, the results were not validated in animal or tissue experiments. Moreover, more experiments should be performed in future work to confirm and illustrate the detailed functional roles of the dysregulated lncRNAs by using RNA interference approaches in vitro and in vivo.

## Conclusions

In conclusion, this is the first report of microarray analysis of lncRNA and mRNA differential expression in LECs during TGF-β_2_-induced EMT compared to normal LECs. These differentially expressed lncRNAs and mRNAs likely play important roles in the development and progression of EMT in LECs. Our study lays the foundation for future research on lncRNAs related to EMT in LECs and could introduce new avenues for the prevention and treatment of PCO.

## Abbreviations

EMT: Epithelial-mesenchymal transition
GO: Gene ontology
HLE B-3: Human lens epithelial B-3
KEGG: Kyoto Encyclopedia of Genes and Genomes
LECs: Lens epithelial cells
lncRNAs: Long noncoding RNAs
PCO: Posterior capsule opacification
qRT-PCR: Quantitative real-time reverse transcription polymerase chain reaction
TGF-β_2_: Transforming growth factor β_2_
